# Integration of expression data in genome-scale metabolic network reconstructions

**DOI:** 10.3389/fphys.2012.00299

**Published:** 2012-08-06

**Authors:** Anna S. Blazier, Jason A. Papin

**Affiliations:** Department of Biomedical Engineering, University of Virginia, CharlottesvilleVA, USA

**Keywords:** flux balance analysis, data integration, transcriptomics, expression data, metabolic networks

## Abstract

With the advent of high-throughput technologies, the field of systems biology has amassed an abundance of “omics” data, quantifying thousands of cellular components across a variety of scales, ranging from mRNA transcript levels to metabolite quantities. Methods are needed to not only integrate this omics data but to also use this data to heighten the predictive capabilities of computational models. Several recent studies have successfully demonstrated how flux balance analysis (FBA), a constraint-based modeling approach, can be used to integrate transcriptomic data into genome-scale metabolic network reconstructions to generate predictive computational models. In this review, we summarize such FBA-based methods for integrating expression data into genome-scale metabolic network reconstructions, highlighting their advantages as well as their limitations.

## Introduction

A central challenge in the development of systems biology is the integration of high-throughput data to generate predictive computational models. With the advent of high-throughput technologies, “omics” data types have provided quantitative data for thousands of cellular components across a variety of scales. Genomics provides data on a cell's DNA sequence, transcriptomics on the mRNA expression of cells, proteomics on a cell's protein composition, and metabolomics on a cell's metabolite abundance. Computational methods are needed to reduce this dimensionality across the wide spectrum of omics data to improve understanding of the underlying biological processes (Cakir et al., 2006; Pfau et al., 2011).

Metabolic network reconstructions are an advantageous platform for the integration of omics data (Palsson, 2002). Assembled in part from annotated genomes as well as biochemical, genetic, and cell phenotype data, a metabolic network reconstruction is a manually-curated, computational framework that enables the description of gene-protein-reaction relationships (Chavali et al., 2012). Numerous studies have demonstrated how such reconstructions of metabolism can guide the development of biological hypotheses and discoveries (Oberhardt et al., 2010; Sigurdsson et al., 2010; Chang et al., 2011).

Flux balance analysis (FBA), a constraint-based modeling approach, can be used to probe these network reconstructions by predicting physiologically relevant growth rates as a function of the underlying biochemical networks (Gianchandani et al., 2009). To do so, FBA involves delineating constraints on the network according to physicochemical, environmental, regulatory, and thermodynamic principles (Kauffman et al., 2003; Price et al., 2003). After applying constraints, the solution space of possible phenotypes narrows, allowing for more accurate characterization of the reconstructed metabolic network. Omics data can be used to further constrain the possible solution space and enhance the model's predictive powers (Palsson, 2002; Lewis et al., 2012).

Given the wealth of transcriptomic data, efforts to integrate mRNA expression data with metabolic network reconstructions, have, in particular, made significant progress when using FBA as an analytical platform (Covert and Palsson, 2002; Akesson et al., 2004; Covert et al., 2004). However, despite this abundance of data, the integration of expression data faces unique challenges such as experimental and inherent biological noise, variation among experimental platforms, detection bias, and the unclear relationship between gene expression and reaction flux (Zhang et al., 2010). Nevertheless, the past few years have witnessed several advances in the integration of transcriptomic data with genome-scale metabolic network reconstructions. Specifically, numerous FBA-driven algorithms have been introduced that use experimentally derived mRNA transcript levels to modify the network's reactions either by inactivating them entirely or by constraining their activity levels. Such algorithms have demonstrated their applicability by, for example, classifying tissue-specific metabolic activity in the human network and by identifying novel drug targets in *Mycobacterium tuberculosis* (Shlomi et al., 2008; Colijn et al., 2009).

In this review, we will first give an overview of the formulation of FBA. Subsequently, we will summarize various FBA-driven methods for integrating expression data into genome-scale metabolic network reconstructions. Finally, we will survey the limitations of these algorithms as well as look to the future of multi-omics data integration using genome-scale metabolic network reconstructions as the scaffold.

## Flux balance analysis

FBA is a constraint-based modeling approach that characterizes and predicts aspects of an organism's metabolism (Gianchandani et al., 2009) To use FBA, the user supplies a metabolic network reconstruction in the form of a stoichiometric matrix, **S**, where the rows in **S** correspond to the metabolites of the reconstruction and the columns in **S** represent reactions in the reconstruction. For each matrix element, a stoichiometric coefficient **s**_**ij**_ conveys the molecularity of a certain metabolite in a particular reaction, with **s**_**ij**_ ≥ 1 indicating that the metabolite is a product of the reaction, **s**_**ij**_ ≤ −1 a reactant, and **s**_**ij**_ = 0 signifies that the metabolite is not involved.

Subsequently, a system of linear equations is established by multiplying the **S** matrix by a column vector, **v,** which contains the unknown fluxes through each of the reactions of the **S** matrix. Under the assumption that the system operates at steady-state, that is to say there is no net production or consumption of mass within the system, the product of this matrix multiplication must equal zero, **S** · **v** = 0 (Gianchandani et al., 2009). Because the resulting system is underdetermined (i.e., too few equations, too many unknowns), linear programming (LP) is used to optimize for a particular flux, **Z**, the objective function, subject to underlying constraints. The objective function typically takes on the form of:

Z=c⋅v

where **c** is a row vector of weights for each of the fluxes in column vector **v**, indicating how much each reaction in **v** contributes to the objective function, **Z** (Lee et al., 2006; Orth et al., 2010). Examples of objective functions include maximizing biomass, ATP production, and the production of a metabolite of interest (Lewis et al., 2012). The following linear program is an example of how FBA problems are formulated:

(1) $$\begin{array}{cc} \text{maximize} & \text{Z}=\text{c}\cdot \text{v} \end{array}$$

subject to

(2) S⋅v=0

(3) lb≤v≤ub

where (1) outlines the objective function to be optimized, (2) the steady state assumption, and (3) describes the upper and lower bounds, *ub* and *lb*, of each of the fluxes in **v** according to such constraints as enzyme capacities, maximum uptake and secretion rates, and thermodynamic constraints (Price et al., 2003; Jensen and Papin, 2011). Through this application of constraints, the solution space of physiologically feasible flux distributions for **v** shrinks. Thus, the task of FBA is to find a solution to **v** that lies within the bounded solution space and that optimizes the objective function at the same time.

## Algorithms for the integration of expression data

**Figure 1:**
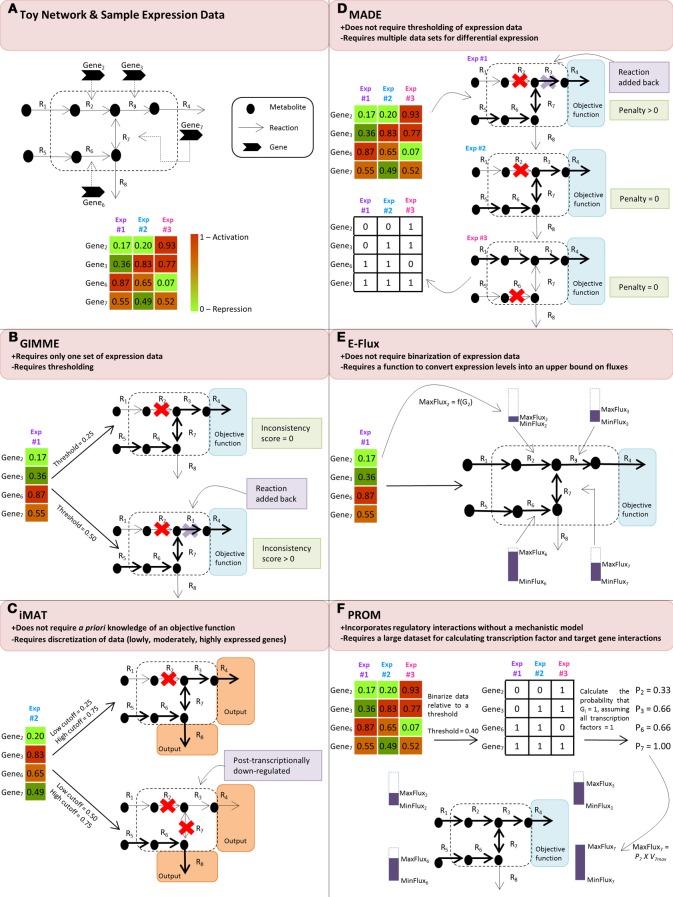
**(A)** Toy network and sample expression data for three different experimental conditions. Panels **(B)**, **(C)**, **(D)**, **(E)** and **(F)** demonstrate how the discussed algorithms use the sample expression data to modify the flow of reaction flux, shown in the boldness of the reaction arrow, through the toy network. **(B)** GIMME compares the expression data to a threshold and subsequently removes reactions whose expression levels fall below the threshold. In the second case where the threshold is 0.50, the removal of R_3_ causes the network to not achieve its user-specified objective function, resulting in a non-functional model; thus, it is added back into the network to create a functional model. **(C)** iMAT discretizes the expression data into lowly, moderately, and highly expressed reaction sets and removes reactions that are lowly expressed from the network. Because the network is not required to achieve a specified objective function, both cases of thresholding result in a functional model. However, in the second case where the low cutoff is 0.50 and the high cutoff is 0.75, R_3_ is considered to be post-transcriptionally down-regulated because, even though expression data shows it to be highly expressed, the resulting toy network indicates that R_3_ has little, if any, reaction flux. **(D)** MADE determines a sequence of binary expression states by maximizing the statistically significant changes across the expression states and simultaneously solves the flux balance analysis problem for each of the experimental conditions, resulting in unique toy networks for each condition. **(E)** E-Flux uses the expression data to modify the maximum possible flux, MaxFlux_i_, through the relevant reactions. **(F)** PROM first binarizes the data according to a threshold to determine on/off states and then determines the probability that a gene is on when its transcription factor is also on. In this example, we assume that the transcription factors are on for all of the genes. Using this probability, PROM modifies the MaxFlux_i_ through the relevant reactions.

Several recently developed algorithms have demonstrated how expression data can be incorporated into FBA models to further constrain the flux distribution solution space in genome-scale metabolic network reconstructions (Figure 1; Table 1). The following section highlights the theory behind several of these methods as well as key differences in their structure.

**Table 1:** **Summary of the algorithms for the integration of expression data**.

**Method**	**Description**	**Advantages**	**Disadvantages**	**Reference**
GIMME	Compares expression levels to a threshold to determine sets of active reactions and sets of inactive reactions in a reconstruction and returns a functioning model that meets an assumed objective function	Only requires one gene expression data set	Requires thresholding of mRNA transcript levels relative to a user-specified value	Becker and Palsson, 2008
iMAT	Uses expression data to determine highly active, moderately active, and lowly active sets of reactions in the reconstruction and solves a MILP problem to return a functioning model	Does not require *a priori* knowledge of metabolic functionalities	Requires discretization of expression data into lowly, moderately and highly expressed genes	Shlomi et al., 2008
MADE	Requires two or more sets of microarray data to create a sequence of binary expression states for a reconstruction's reactions, removing the need for arbitrary thresholding	Does not require a user-supplied threshold to determine which reactions are highly expressed and which are lowly expressed	Requires multiple datasets for differential expression	Jensen and Papin, 2011
E-Flux	Compares expression levels to a threshold and subsequently constrains the upper bounds of the reactions that are lowly expressed	Does not reduce gene expression data to binary on-off states	Requires a function to convert expression levels into an upper bound on fluxes	Colijn et al., 2009
PROM	Determines the probability that a gene is active relative to the activity of its transcription factor according to expression data and subsequently constrains the maximum flux for relevant reactions by a factor of this probability	Incorporates regulatory interactions without a mechanistic model	Requires a large dataset for calculating transcription factor and target gene interactions	Chandrasekaran and Price, 2010

### GIMME

One such method that guarantees to both produce a functioning metabolic model based on gene expression levels and quantify the agreement between the model and the data is called the Gene Inactivity Moderated by Metabolism and Expression (GIMME) algorithm (Becker and Palsson, 2008). To execute this algorithm, the user supplies a set of gene expression data, a genome-scale metabolic network reconstruction, and at least one specified metabolic functionality, or objective function, that the cell is assumed to achieve. Broken into two steps, the GIMME algorithm first runs FBA on the original reconstruction to find the maximum possible flux through the specified functionalities. Subsequently, under the assumption that gene expression data correlate with reaction fluxes, GIMME compares the experimental mRNA transcript levels to a user-specified threshold and methodically removes reactions from the model whose mRNA transcript levels fall below the given threshold. However, in the event that the resulting model is unable to achieve the desired objective function, GIMME solves an LP problem that adds sets of the inactive reactions back into the system in such a way that minimizes deviation from the expression data. To quantify this deviation, an inconsistency score is calculated for each reaction by multiplying the flux necessary to achieve a defined value of the objective function and the distance between the experimental mRNA transcript level and the threshold level. By minimizing this inconsistency score, GIMME uses expression data to define a functioning metabolic model that meets the assumed functionality and is consistent with the experimentally measured gene expression data.

### iMAT

Similar to GIMME, the Integrative Metabolic Analysis Tool (iMAT) results in a functioning model in which the fluxes of reactions correlated with high mRNA levels are maximized and the fluxes of reactions associated with low mRNA levels are minimized (Shlomi et al., 2008; Zur et al., 2010). A key difference is that iMAT does not require *a priori* knowledge of a defined metabolic functionality. Briefly, this method establishes a tri-valued gene-to-reaction mapping for each reaction in the model according to the level of gene expression in the data. More specifically, highly, moderately, and lowly expressed genes relative to a user-specified threshold are assigned values of 1, 0, and −1, respectively, resulting in sets of highly expressed reactions (*R*_H_) and sets of lowly expressed reactions (*R*_L_). The algorithm then solves a mixed integer linear programming (MILP) problem according to stoichiometric and thermodynamic constraints while also maximizing *R*_H_ and minimizing *R*_L_. Mirroring GIMME, the presence of reactions is allowed to deviate from the expression data in order for the algorithm to result in a functioning model. Thus, like GIMME, iMAT returns a functioning metabolic model that integrates expression data with a genome-scale metabolic network reconstruction by maximizing the number of highly expressed reactions and minimizing the number of lowly expressed reactions; however, unlike GIMME, this method does not demand that the user supply functionalities that the model is assumed to meet. Instead, iMAT requires that reactions catalyzed by the products of highly expressed genes are able to carry a minimum flux. By removing this need for user-specified objective functions, iMAT bypasses assumptions about metabolic functionalities of a particular network, which proves advantageous for models where there is no clear objective function, as in models of mammalian cells.

### MADE

While both GIMME and iMAT rely on user-specified threshold values to determine which reactions are highly expressed and which reactions are lowly expressed, Metabolic Adjustment by Differential Expression (MADE) uses statistically significant changes in gene expression measurements to determine sequences of highly and lowly expressed reactions (Jensen and Papin, 2011). In order for MADE to generate these expression states, the user must supply expression data from two or more experimental conditions. For each gene, the algorithm calculates the changes in the mean expression level across the conditions and returns a sequence of differences, according to an increase (+1), decrease (−1) or no change (0) in mRNA levels. MADE then determines each gene's sequence of binary expression states across the experimental conditions by finding the pattern of changes that most closely mirrors the sequence of differences. While determining this sequence of binary states, MADE simultaneously solves the FBA problem for each condition, resulting in a sequence of functioning models. The statistical significance of the expression changes is used as a weighting to resolve conflicts between expression changes and model functionality. MADE's reliance on statistically significant changes across various experimental conditions to constrain reaction activity avoids the arbitrariness surrounding the determination of a proper threshold in prior methods. As will be discussed in more detail later, the lack of correlation between mRNA levels and protein levels makes it difficult to accurately determine when genes are “turned on,” and when they are “turned off.” Therefore, in eliminating this need for thresholding, MADE removes significant user-bias from the system.

### E-Flux

Whereas GIMME, iMAT, and MADE incorporate gene expression data into their models by reducing gene expression levels to binary states, the method E-Flux attempts to more directly incorporate gene expression data into FBA optimization problems by constraining the maximum possible flux through the reactions (Colijn et al., 2009). Rather than setting the upper bounds of a reaction to some large constant or 0, mirroring the implementation of binary-based algorithms, E-Flux constrains the upper bound of a reaction according to its respective gene expression level relative to a particular threshold. In cases where the gene expression data is below a certain threshold, tight constraints are placed on the flux through the corresponding reactions in the reconstruction; conversely, in cases where the gene expression is above a certain threshold, loose constraints are placed on the flux through the corresponding reactions. The width of this “flux cone” is adjusted by changing the maximum possible flux in the upper bound of the FBA optimization problem according to some function of the gene expression level. While the other algorithms described above use mRNA expression data to predict binary states for the network reactions, E-Flux uses the transcript levels to determine the degree to which a reaction is active or inactive. In doing so, E-Flux offers a more physiologically relevant depiction of the continuous nature of the reaction activity gradient.

### PROM

In contrast to the other methods discussed, which focused solely on integrating gene expression data into genome-scale metabolic network reconstructions, Probabilistic Regulation of Metabolism (PROM) aims to fuse together metabolic networks and transcription regulatory networks with expression data (Chandrasekaran and Price, 2010). To run PROM, the user supplies a genome-scale metabolic network reconstruction, a regulatory network structure describing transcription factors and their targets, and a range of expression data from various environmental and genetic perturbations. Given this expression data, PROM binarizes the genes with respect to a user-supplied threshold to evaluate the likelihood of the expression of a target gene given the expression of that gene's transcription factor. For example, a probability of 0.6 for gene X when its transcription factor Y is on represents that in 60% of the microarray samples when transcription factor Y is highly expressed, gene X exhibits high expression. Upon calculating these probabilities, PROM then incorporates them into FBA by constraining the upper bound to *P* × *V*_*max*_, where *P* is the probability that the gene is on for that reaction and *V*_*max*_ is the maximum flux through that reaction. Therefore, although PROM does binarize the data to calculate the probabilities of gene activity, the actual integration of the expression data into the FBA model does not reduce the reactions to on/off states; rather, similarly to E-Flux, PROM constrains the maximum flux through relevant reactions according to transcription factor activity.

## Challenges facing the integration of expression data

Each of the methods discussed hinges on the assumption that mRNA transcript levels are a strong indicator for the level of protein activity. For instance, GIMME and iMAT assume that mRNA levels below a certain threshold suggest that the corresponding reactions are inactive. MADE follows a similar logic, turning reactions on and off depending on the changes in mRNA transcript levels. E-Flux and PROM assume that transcript levels indicate the degree to which reactions are active, evident in the constraining of the upper bounds in the FBA optimization problems associated with these methods.

However, recent studies have called into question the validity of this assumption which correlates mRNA transcript levels to protein levels. In one of the first large-scale screens to assess the correlation between mRNA and protein expression, the Pearson product moment correlation coefficient, a measure of the linear dependence between two variables ranging from −1 to 1, was calculated to be 0.356 for yeast data (Gygi et al., 1999). Subsequently, several groups have also tried to assess this relationship between mRNA and protein levels by calculating the Spearman rank correlation, an analogue of the Pearson product moment correlation coefficient using ranked variables. With values ranging from 0.21 to 0.61, this positive correlation between mRNA abundance and protein abundance is moderate at best (Ideker et al., 2001; Griffin et al., 2002; Washburn et al., 2003; Moxley et al., 2009). Possible reasons for this discrepancy include post-translational modifications, post-transcriptional regulation and enzyme kinetics (Zhang et al., 2010). Interestingly, a recent study demonstrated that the level of transcriptional regulatory activity on observed protein expression varies from pathway to pathway according to an evolutionary trade-off between minimizing protein cost and rapidly responding to environmental changes (Wessely et al., 2011).

Acknowledging this questionable association between transcriptomic and proteomic data, the discussed methods claim to treat the assumption that mRNA levels serve as a strong indicator for protein activity as a “soft constraint.” Rather than requiring that the reconstruction mirror the expression data exactly, the methods allow for deviations in the FBA flux solution space in order to generate a functioning model that adheres to the specified constraints. In the case of GIMME, highly expressed reactions are prioritized relative to lowly expressed reactions; however, in the event that an optimal, functioning solution cannot be found, the assumption can be violated and lowly expressed reactions can be added back into the reconstruction. Thus, this assumption that mRNA transcript levels correlate to protein levels serves as a cue rather than a mandate.

## Conclusion

The above methods have been used to not only integrate expression data from a variety of sources but to also make progress toward overcoming key challenges in the field of systems biology. For instance, iMAT, highlighting its applicability in multi-cellular organisms, was used to curate the human metabolic network reconstruction and predict tissue-specific gene activity levels in ten human tissues (Duarte et al., 2007; Shlomi et al., 2008). Additionally, both E-Flux and PROM have been used to discover novel drug targets in *Mycobacterium tuberculosis* (Colijn et al., 2009; Chandrasekaran and Price, 2010).

Given the recent success with using genome-scale metabolic network reconstructions as a platform for integrating expression data, efforts should focus on multi-omics data integration. A handful of methods have already been introduced that integrate two or more types of omics data into genome-scale metabolic network reconstructions. For example, despite the current dearth of quantitative metabolomics data, a method has been developed that demonstrates how semi-quantitative metabolomics data can be used with transcriptomic data to curate genome-scale metabolic network reconstructions and identify key reactions involved in the production of certain metabolites (Cakir et al., 2006). Another algorithm, called Integrative Omics-Metabolic Analysis (IOMA), integrates metabolomics data and proteomics data into a genome-scale metabolic network reconstruction by evaluating kinetic rate equations subject to quantitative omics measurements (Yizhak et al., 2010). Furthermore, Mass Action Stoichiometric Simulation (MASS) uses metabolomic, fluxomic, and proteomic data to transform a static stoichiometric reconstruction of an organism into a large-scale dynamic network model (Jamshidi and Palsson, 2010). And finally, building off of iMAT, the Model-Building Algorithm (MBA) utilizes literature-based knowledge, transcriptomic, proteomic, metabolomic, and phenotypic data to curate the human metabolic network reconstruction to derive a more complete picture of tissue-specific metabolism (Jerby et al., 2010). Such algorithms show promise in their ability to easily integrate high-throughput data into genome-scale metabolic network reconstructions to generate phenotypically accurate and predictive computational models.

### Conflict of interest statement

The authors declare that the research was conducted in the absence of any commercial or financial relationships that could be construed as a potential conflict of interest.
